# Microglial Immune Response to Low Concentrations of Combustion-Generated Nanoparticles: An In Vitro Model of Brain Health

**DOI:** 10.3390/nano8030155

**Published:** 2018-03-09

**Authors:** Cayla M. Duffy, Jacob Swanson, William Northrop, Joshua P. Nixon, Tammy A. Butterick

**Affiliations:** 1 Minneapolis Veterans Affairs Health Care System, Minneapolis, MN 55417, USA; duffy124@umn.edu (C.M.D.); nixon049@umn.edu (J.P.N.); 2 Department of Food Science and Nutrition, University of Minnesota, St. Paul, MN 55108, USA; 3 Department of Integrated Engineering, Minnesota State University, Mankato, MN 56001, USA; jacob.swanson@mnsu.edu; 4 Department of Mechanical Engineering, University of Minnesota, Minneapolis, MN 55455, USA; wnorthro@umn.edu

**Keywords:** combustion-generated nanoparticles, pollution, microglia, chronic inflammation, in vitro biosensor, neuroinflammation

## Abstract

The brain is the central regulator for integration and control of responses to environmental cues. Previous studies suggest that air pollution may directly impact brain health by triggering the onset of chronic neuroinflammation. We hypothesize that nanoparticle components of combustion-generated air pollution may underlie these effects. To test this association, a microglial in vitro biological sensor model was used for testing neuroinflammatory response caused by low-dose nanoparticle exposure. The model was first validated using 20 nm silver nanoparticles (AgNP). Next, neuroinflammatory response was tested after exposure to size-selected 20 nm combustion-generated nanoparticles (CGNP) collected from a modern diesel engine. We show that low concentrations of CGNPs promote low-grade inflammatory response indicated by increased pro-inflammatory cytokine release (tumor necrosis factor-α), similar to that observed after AgNP exposure. We also demonstrate increased production of reactive oxygen species and nuclear factor kappa-light-chain-enhancer of activated B cells (NF-κB) p65 phosphorylation in microglia after CGNP stimulation. Finally, we show conditioned media from CGNP-stimulated microglia significantly reduced hypothalamic neuronal survival in vitro. To our knowledge, this data show for the first time that exposure to AgNP and CGNP elicits microglial neuroinflammatory response through the activation of NF-κB.

## 1. Introduction

Environmental pollution from anthropogenic combustion sources negatively affects the health of millions of people worldwide. Combustion-generated (CG) emissions include a complex mixture of volatile organic gases, carbon monoxide, oxides of nitrogen, and other solid and semi-volatile particulate matter (PM). Engine emissions from on and off-road diesel-powered vehicles and jet aircraft are a major source of combustion-generated pollution, contributing in total about 50% of all black carbon (BC) emissions in the US [1,2]. Although most new diesel engines in the US are fitted with diesel particulate filters that remove greater than 99% of PM, legacy diesel engines produced before 2007 do not have filters and jet aircraft cannot have post-combustion emissions; thus, PM emissions will continue to have impacts on health and the environment well into the future [3].

Combustion-generated PM is found primarily in three primary size modes categorized according to diameter [4]: the coarse mode in the range of 500–10,000 nm; the accumulation mode in the range of 30–500 nm consisting of mainly of solid elemental carbon agglomerates (a surrogate for BC) with adsorbed organic material; and the nucleation mode in the range of 5–50 nm consisting of mainly semi-volatile organic compounds, like aliphatic and aromatic hydrocarbons, including polycyclic aromatics, sulfates, and traces of solid metallic ash. The nanoparticle (NP) range of PM is generally considered to be particles with diameter less than 50 nm [5]. The solid NP fraction mostly consists of nuclei mode metallic ash components derived from metals in the lubricating oil, including calcium, zinc, phosphorus, iron, manganese, and others, as well as small carbon agglomerates from the accumulation mode [6].

The main route of PM infiltration is through respiration with deposition being dependent on particle size [7,8,9,10]. The total deposition efficiency for PM is primarily a function of size, particles with diameter greater than 1 μm are deposited through inertial impaction. Particles smaller than 1 μm remain in the gas flow and are deposited in the thoracic tree or lung. Those particles with diameters <50 nm (NPs) have a larger deposition rate in the alveolar region. They also have increased deposition in the nasal cavity due to diffusion, with particles <10 nm attaining 20–80% deposition efficiency [7,8,10].

Recently, the International Agency for Research on Cancer (IARC), an intergovernmental agency forming part of the World Health Organization classified PM from diesel engines as carcinogenic to humans (Group 1), clearly indicating consensus on its adverse human health effect [5,11,12]. Previous investigations in both human and rodent models evaluating PM exposure have predominately focused on linkages to increased cancer risk and adverse cardiovascular and respiratory effects, but mounting data supports adverse endocrine and brain effects [11,12,13,14,15,16,17,18,19,20]. Prior toxicology studies have demonstrated that 20 nm diameter AgNP increase cellular toxicity, inflammation, and oxidative stress in rodent and hepatocyte models [21,22,23]. It has also been demonstrated that, in addition to the onset of peripheral and neuroinflammation, AgNP within the 20–25 nm diameter range can gain access to brain tissue via altering BBB permeability, inducing neurotoxicity, motor deficits, and cognitive decline [21,22,24,25,26,27,28].

Although the respiratory and cardiovascular system impacts of combustion-generated particulate matter (PM) are well established, the long-term effect of PM on brain health has not been thoroughly studied [18,19,29,30]. Recent research suggests that some metal elements found in PM emissions may act as neurotoxins [31,32]. Elevated exposure to PM may induce neuroinflammation and influence progression of neurodegenerative diseases, such as Alzheimer’s or Parkinson’s disease [13,30,33]. The brain is normally protected against environmental factors, such as PM, by the blood brain barrier (BBB), a selectively permeable biological “firewall” that restricts passage of substances into the brain. However, nanoparticle (NP)-sized PM components have been found to breach the BBB and directly enter the central nervous system CNS [13,14,28,34,35]. These NP-sized PM components, known as combustion-generated nanoparticles (CGNP), are generated by combustion sources, such as internal combustion engines, aircraft turbine engines, and coal-based power generation plants [36,37,38]. Particles in the NP size range pose a concern for adverse effects on CNS health due to their ability to migrate through the BBB and enter the brain [28,35]. The primary routes of environmental NP infiltration are through nasal respiration, via direct conduction along the olfactory nerve, or via alveolo-capillary translocation [28,35]. Particles have increased deposition in the nasal cavity with decreased size due to diffusion, with particles smaller than 10 nm attaining 20–95% deposition efficiency in the nasal cavity [34]. CGNPs with diameters of 20 nm or smaller have a high deposition efficiency along the respiratory track, and short-term chronic exposure to CGNPs in this size range is known to negatively impact brain health [13,33,35,39,40]. CGNPs thus represent a direct link between PM and brain health.

Previous investigations in human and rodents evaluating the impact of CGNP exposure have predominately focused on linkages to increased cancer risk and adverse cardiovascular, respiratory, and endocrine effects [13,41]. While increased exposure to air pollution, particularly CGNP, has been shown to induce disorders of the CNS, the mechanisms are poorly defined [13]. The onset of neurotoxicity can directly alter neuronal and astroglial cell function, depending on factors as duration of exposure, size, shape, and composition of particles [24]. However, these studies do suggest that the neuroinflammatory response is a key component. Upon contact with brain tissue, NPs can induce the onset of neuroinflammation via activation of microglia (brain immune cells) and neurotoxic phenotype [24,28]. Neuroinflammation within the hippocampus has been previously indicated in neurodegeneration and subsequent cognitive decline [21,42,43]. Epidemiological data and rodent models correlate air pollution levels to increased memory impairment, risk for Alzheimer’s and Parkinson’s disease, particularly in at-risk populations, such as the elderly and/or socioeconomically disadvantaged populations [33,42,44,45]. These data collectively support that environmental exposure to CGNPs can directly impair normal brain function.

To date, determining detrimental effects of CGNP in vivo has required time-consuming and expensive methodologies [46]. Furthermore, the complex nature of CGNP makes screening of individual components using in vivo models cumbersome. Thus, the risk CGNPs pose to human brain health has remained relatively unexplored. With no known standardized screening methods, it is imperative to develop an economical biosensor that can rapidly test the neurotoxicity of CGNPs in the brain. Microglia, the resident immune cells in the brain, mediate the brain’s inflammatory response to neural insults. These characteristics make microglia an ideal in vitro biosensor surrogate to study the neurotoxicity of air pollutants and to definite mechanisms that elicit neurotoxic responses to environmental exposures.

**Figure 1:**
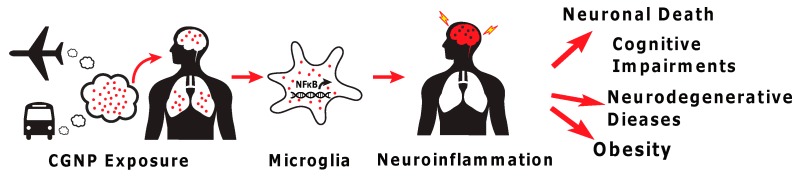
Central hypothesis. Nanoparticle exposure causes deleterious effects on brain health through activation of microglia, triggering low-grade chronic neuroinflammation and subsequent dysregulation of brain function.

Our overall hypothesis is that CGNP exposure causes deleterious effects on brain health through activation of microglia, triggering low-grade chronic neuroinflammation and subsequent dysregulation of brain function, leading to various downstream health consequences, such as cognitive impairment (Figure 1). Our aim is to test low-dose chronic exposure to NP, rather than acute apoptotic response to higher NP concentrations (>0.5 μg/mL) used by others, as these higher acute doses may not reflect the ambient environmental exposure observed in real-world settings [20,29,47,48]. Here we first validate our in vitro model by exposing microglia to low concentrations of 20 nm silver NP (AgNP) by profiling immune response, specifically the release of neurotoxic pro-inflammatory cytokines and the activation of NFκB, the upstream regulator of immune response [21]. To test our model using a real-world exposure paradigm, CGNPs were generated from a diesel compression ignition engine and microglial immune response to CGNPs was characterized and compared to the response from exposure to AgNPs.

## 2. Materials and Methods

### 2.1. Cell Culture and Reagents

Cell culture conditions were described previously [49]. Briefly, immortalized murine microglial cells (BV2) [50] and adult murine hypothalamic cells (mHypoA-1/2; CELLutions Biosystems, Burlington, NC, USA) were grown in Dulbecco’s modified Eagle’s medium (DMEM; Thermo Fisher Scientific, Waltham, MA, USA) plus 10% fetal bovine serum and 1% penicillin, streptomycin, and neomycin (PSN; Thermo Fisher) and maintained at 37 °C with 5% CO_2_. Commercially purchased and standardized silver nanoparticles (AgNP, average 20 nm ± 4 nm, as quality controlled by spectrometric analysis at 390–410 nm and TEM imaging; suspended in 2 μM sodium citrate solution; Sigma-Aldrich, St. Louis, MO, USA) were diluted in DMEM plus 1% PSN to desired concentrations (0.01, 0.05, 0.1, 1, 5, 10 μg/mL). Lipopolysaccharide (LPS; Sigma-Aldrich) was reconstituted in PBS and diluted to 0.1 μg/mL in DMEM. BV2 cells were seeded in T-75 cell culture plates (Sigma-Aldrich) and grown until 80% confluent. Cells were then reseeded in six-well plates at ~3.8 × 10^4^ cells/well or in 96-well plates at ~8 × 10^3^ cells/well overnight then serum-starved for 24 h before challenge (NP, controls). For the challenge, cells were incubated with vehicle control (sodium citrate), AgNP, CGNP (0.01 μg/mL), or LPS (0.1 μg/mL, positive control). The supernatant was collected immediately after challenge, then immediately filtered and stored at −20 °C.

### 2.2. Combustion-Generated Nanoparticles

Combustion-generated particles were generated using a modern 2.0 L, 117 kW turbocharged, compression ignition engine (General Motors, Detroit, MI, USA). This engine is factory equipped with a variable geometry turbocharger, variable swirl actuation, high pressure common rail fuel injection, and exhaust gas recirculation. It was fueled with ultra-low sulfur diesel fuel (ULSD). Exhaust was diluted using a single-stage ejector dilutor with a dilution ratio of 10:1. CGNP particles were size-selected using a scanning differential mobility analyzer (DMA), which selects particles based on their “electrical mobility” diameter. For very small particles, the electrical mobility diameter is nearly the same as the geometric diameter and hydrodynamic diameter. For example, Fissan et al. showed that 18.3 nm Ag-Au particles (determined by scanning electron microscopy, SEM) had a 22 nm hydrodynamic diameter and 22 nm electrical mobility diameter [51]. To collect particles for exposure, a sample was taken from the dilute exhaust stream, passed through a catalytic stripper to remove semi-volatile material, and then size-selected to 20 nm using a long-column TSI (Shoreview, MN, USA) 3081 DMA [36,52]. CGNP particles were size-selected using a scanning differential mobility analyzer, which selects particles based on their “electrical mobility” diameter. For very small spherical particles, the electrical mobility diameter is nearly the same as the geometric diameter and hydrodynamic diameter [53].

Particles were collected by passing the aerosol through a silver membrane filter with an efficiency of 100%. During collection, the particle concentration was monitored with a TSI 3776 condensation particle counter in parallel with the filter to determine the total collected mass. The upstream solid particle concentration was approximately 10,000 part/cm^3^. Silver filters were placed in a vial containing sodium citrate submerged in an ultrasonic bath to separate the particles from the filter. The bath was operated at 35 °C for 30 min. The particle concentration variation was less than 5%/h. The zeta potential of CGNPs were not measured in this work, but the others have measured the zeta voltages for diesel particles suspended in water and found values of −48 mV to −31 mV [54,55]. 

### 2.3. Cell Toxicity Assay

Media supernatant collected after NP challenges was used to determine lactate dehydrogenase (LDH) cytotoxicity activity according to manufacturer’s protocol (Cayman Chemical, Ann Arbor, MI, USA). Briefly, media supernatant was collected after NP treatment and filtered using a 0.22 μm sterile filter. To measure LDH activity, 100 μL of the reaction mix was added to a clear bottom 96-well plate with 100 μL of filtered media supernatant and incubated at room temperature for 30 min. Absorbance was then measured at 490 nm using a spectrophotometer (SpectraMax-M5; Molecular Devices, San Jose, CA, USA). Results are presented as percent LDH release vs. maximum LDH release.

### 2.4. Cell Metabolic Assay

Cell metabolic activity measures were used to demonstrate microglia activation. Activity was determined using a resazurin-based assay (PrestoBlue, Thermo Fisher) as previously described [21,56]. This assay can detect shifts in the redox status within a cell and reflects the metabolic conversion rate and altered cellular metabolism that is correlated with an increase in pro-inflammatory M1-like microglia and is non-specific to electron transport or mitochondrial function [57]. The shift in oxidized to reduced states allows for the detection of metabolically-activated microglia that are able to reduce the blue resazurin (non-fluorescent) compound to resorufin (a highly fluorescent) compound [58]. Briefly, 10 μL of resazurin reagent was added to each well, containing 90 μL of medium. Cells were incubated for 10 min at 37 °C post-NP or LPS challenge. Fluorescence (excitation/emission, 560/590 nm) was measured using a spectrophotometer. Results are presented as the change in relative fluorescence units (RFU) vs. control [21].

### 2.5. Enzyme-Linked Immunosorbent Assay (ELISA)

Media was first filtered to remove NP and cellular debris (Amicon Ultra 10k filter; EMD Millipore, Billerica, MA, USA). Secreted tumor necrosis factor-alpha (TNF-α) levels in culture media were determined by using a commercial ELISA kit (BioLegend Inc.; San Diego, CA, USA) per the manufacturer’s instructions and as previously described [49]. Briefly, 100 μL of filtered cell culture media was added to a 96-well plate pre-coated with TNF-α capture antibody in triplicate and incubated at room temperature (RT) in a humidity chamber for 2 h. After washing, secondary antibody was added, and cells were incubated for 1 h at RT. After a second wash, 50 μL of avidin-HRP was added, and plate was incubated for 30 min at RT. Finally, the plate was washed and TMB substrate was added to develop fluorescence for 30 min before stop solution was added. Absorbance was measured at 450 nm using a spectrophotometer (SpectraMax-M5).

The phosphor-RelA/NF-kB p65 (phospho-p65) level in fixed cells was measured using a commercially available in cell-based ELISA kit (R&D Systems, Minneapolis, MN, USA). This assay measures the ratio of phospho-p65 and total p65 levels. BV2 cells were grown and treated in 96-well plates as described in 2.1 and then probed for phosphor- and total-p65 level following NP stimulation. Briefly, cells were fixed in 4% formaldehyde, blocked, and then incubated with primary antibodies provided in the kit, followed by PBS buffer wash and probing with secondary antibodies provided in the kit. Finally, the substrate reagent was added, and the fluorescence of phosphor- and total-p65 were measured using a spectrophotometer (excitation/emission = 540/600 nm). Data are first calculated as the ratio between phosphor-p65 and total-p56 and then presented as the fold change vs. control.

### 2.6. Reactive Oxygen Species (ROS) Assay

Reactive oxygen species (superoxide and hydroxyl radical) were measured using deep red fluorescence (Abcam, Cambridge, MA, USA). BV2 cells grown in 96-well plates were treated with AgNPs and controls (12 wells for each treatment) and then incubated with the deep red stain. Fluorescence (Ex/Em = 650/675 nm) was monitored using a spectrophotometer. The time point was based on a previous report [59]. Results are presented as the fold change vs. control.

### 2.7. Conditioned Media Experiment

Hypothalamic cells (mHypoA-1/2) were seeded in a 96-well plate at 5 × 10^3^ cells per well overnight. Media supernatant taken from microglial cells subjected to NP challenges was first filtered using a filtration device (EMD Millipore) with molecular weight cutoff of 10 kDa, as previously described and validated [21,49]. The purpose of the filtration is to remove the residual NPs left in the media supernatant. Filtrate was then added to the hypothalamic cells and incubated at 37 °C with 5% CO_2_ for 24 h. Cell density (5 × 10^3^ cells per well) and time point (24 h) were determined based on previous studies by our group [49,60]. To determine neuronal cell death, surviving cells were counted by using a trypan blue exclusion test with the aid of an automated cell counter (Countless II FL Automated Cell Counter, Thermo-Fisher). Cell survival is represented as percent of cell survival number relative to the control [49,60].

### 2.8. Statistical Methods

Significant differences were determined by two-sided Student’s *t*-tests using GraphPad Prism 5 (GraphPad Software, La Jolla, CA, USA).

## 3. Results and Discussion

**Figure 2:**
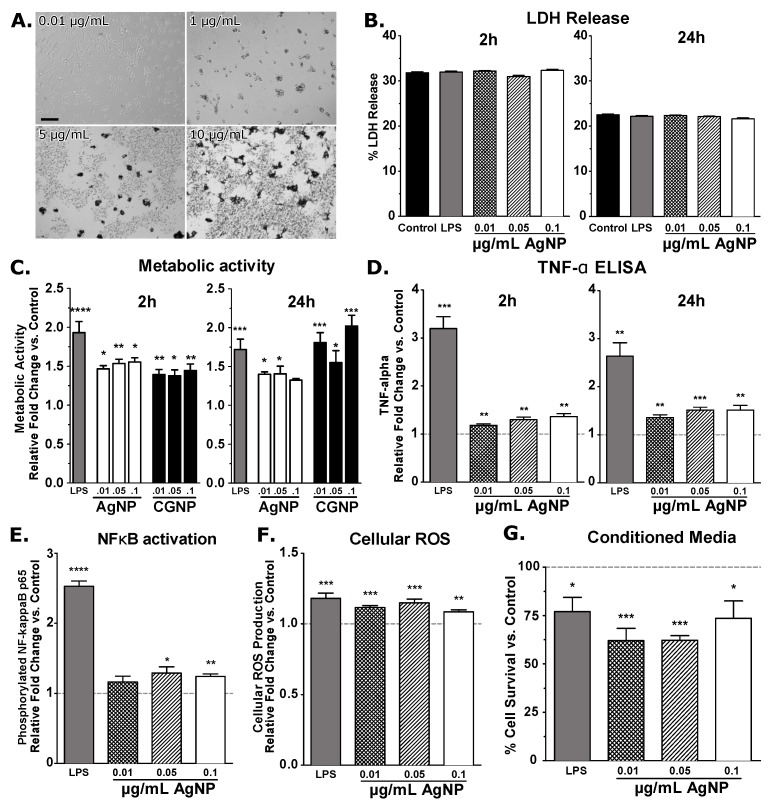
Light microscopic images (20× magnification) (**A**) of 20 nm AgNP with 24 h stimulation at 0.01 μg/mL; 1 μg/mL; 5 μg/mL; 10 μg/mL. Significant particle aggregations can be observed. Scale bar = 100 µm. Lactate Dehydrogenase (LDH) activity (**B**) measured in cell media supernatant did not change after 2 h or 24 h AgNP stimulation, indicating no change in cell death. Metabolic activity (**C**) was measured using resazurin based assay. Both AgNP and DENP increased microglial metabolic activity, not in a dose dependent manner. Tumor necrosis factor alpha (TNF-α) (**D**) levels in cell media increased significantly following 2 h and 24 h AgNP stimulation. Nuclear factor kappa-light-chain-enhancer of activated B cells (NF-κB) (**E**) p65 phosphorylation was increased significantly in microglial cells following 30 min AgNP exosure. Reactive oxygen species (ROS) (**F**) generated in microglia significantly increased after 75 min AgNP stimulation. Hypothalamic cell survival (**G**) neurodegeneration increased (as measured by percent cell survival as compared to control) significantly following 24 h incubation with filtered conditioned media. Student’s *t*-test, * <0.5; ** <0.005; *** <0.001; **** <0.0001 vs. control.

Visible particle and cell aggregation was observed in BV2 cells incubated with AgNP concentrations >1 μg/mL (Figure 2A). A previous study by Singh et al. using macrophages (RAW 264.7) incubated with AgNP > 1 μg/mL showed a significant decrease in cell viability [61]. Another study using intranasal delivery of AgNP in mice demonstrated a transition of AgNPs into multiple brain regions, however aggregated AgNPs were not observed in brain tissue [26]. These data indicate that even in high-dose AgNP exposure, particle aggregation is not observed in vivo, and may not represent real-life exposures or uptake of particles.

Subsequent incubation with 0.01, 0.05, and 0.1 μg/mL AgNP for 2 h and 24 h showed increased metabolic activity (Figure 2C) and increased release of TNF-α (Figure 2D) in a dose-dependent manner, while no change in cell viability was observed by measuring lactate dehydrogenase (LDH) activity in media supernatant (Figure 2B). The phosphor-p65 level in microglia was increased after 30 min stimulation with AgNPs (Figure 2E) indicating the inflammatory response mechanism may be modulated by NF-κB activation, a known master regulator of inflammation. To our knowledge, we are the first to demonstrate that AgNPs may induce microglial inflammatory response through a NF-κB mediated pathway. Reactive oxygen species (ROS) formation in microglia was increased significantly across all concentrations as well (Figure 2F), in agreement with similar time point studies [59]. Conditioned media from AgNP-stimulated microglia significantly decreased hypothalamic cell viability (Figure 2G). Taken together, our data show that low concentrations of AgNP consistently cause low-grade inflammatory response in microglia without causing microglial cell death. This increased inflammatory response has also been shown by others using higher concentrations of AgNPs [61]. Data indicate that the use of AgNP within the 20 nm rage induce pro-inflammatory microglia and represent AgNP as a useful positive control for evaluating NP toxicity screens using microglia.

**Figure 3:**
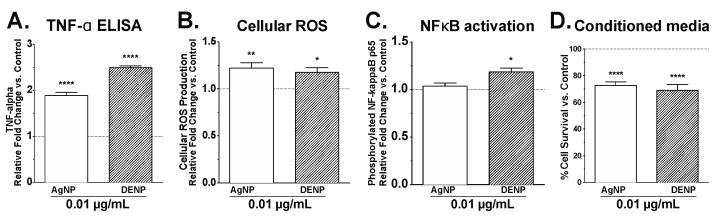
Tumor necrosis factor alpha (TNF-α) (**A**) release into media was significantly increased following CGNP stimulation (labeled as DENP). Reactive oxygen species (ROS) (**B**) generated in microglial cells significantly increased after 75 min stimulation CGNP stimulation (labeled as DENP). Nuclear factor kappa-light-chain-enhancer of activated B cells (NF-κB) (**C**) p65 phosphorylation in microglia was significantly increased after 30 min CGNP stimulation (labeled as DENP) stimulation. Hypothalamic neuronal death (**D**) was increased significantly following 24 h incubation with filtered conditioned media. Student’s *t*-test, * <0.5; ** <0.005; **** <0.0001 vs. control.

In BV2 cells, incubation with 0.01 μg/mL CGNP significantly increased the release of the inflammatory cytokine TNF-α at 24 h post-exposure (Figure 3A). ROS formation (Figure 3B) and NF-κB activation (Figure 3C) were also shown to increase, consistent with that observed following exposure to AgNP. In addition, conditioned media from CGNP-stimulated microglia significantly decreased hypothalamic cell viability (Figure 1D). These data are consistent with that observed in AgNP-treated microglia.

The concentration of CGNP in the ambient air varies depending on the proximity to major roads [62]. It is clear, however, that ambient environmental NPs can enter the central nervous system following inhalation. One study in rats found that between 0.001 and 0.01% of inhaled iridium and carbon NPs can be transported into the brain [63]. If CGNPs have similar deposition efficiency, the concentration used here for microglial studies (0.01 μg/mL CGNP) is equivalent to 100 to 1000 μg/mL or 0.0001 to 0.001 μg/m^3^ in ambient air. Considering the US Environmental Protection Agency set the standard for annual exposure to PM2.5 (particles smaller than 2.5 μm diameter) to be 15 μg/m^3^ for public welfare protection and the short experiment time (24 h), 0.01 μg/mL is unlikely to cause acute apoptotic response and may mimic real-world ambient environmental low-level exposure.

The mild increase in pro-inflammatory cytokines and NF-κB activation support that chronic exposure to low-level ambient CGNPs can potentially result in chronic low-grade neuroinflammation in the brain, a condition which has been shown to trigger cognitive impairment and neurodegeneration, and contribute to neuronal death and serious long-term consequences (Figure 1A) [64]. Due to the important role of the hypothalamus in regulation of energy balance through physical activity and food intake, hypothalamic cell death shown by the conditioned media from CGNP-stimulated microglia supports other prior studies suggesting that chronic exposure to CGNP may also impact energy balance [40,65,66].

Due to the importance of the hypothalamus in regulation of energy balance through physical activity and food intake, hypothalamic cell death shown by the conditioned media from CGNP-stimulated microglia supports prior data suggesting that chronic exposure to CGNP may also impact energy balance [65,66]. Additionally, hypothalamic dysfunction is often reported during the early onset of AD and PD, including non-cognitive deficits related to metabolic symptoms such as mid-life obesity and metabolic syndrome [67,68]. Our model could, thus, assist in the development of rapid biomarker screens to assist in early diagnosis for individuals at risk of neurological insults triggered by environmental pollution exposure [69]. Although our study is limited to testing microglial neurotoxicity using hypothalamic neurons, in future studies, this model can be adapted to evaluate the effects of CGNP on other brain cell types, such as hippocampal or dopaminergic neurons, given their role in AD and PD, respectively.

Various national and international agencies, most notably the World Health Organization (WHO) and US Environmental Protection Agency (EPA), have considered diesel exhaust as a possible carcinogen [3,11,40]. However, NPs typically contain negligible mass and are, thus, not explicitly controlled by US mass-based emissions standards. In addition, while newer engines produce less NPs during use, older engines used in industrial applications generally have decades of longevity, making old-technology diesels likely to have a significant presence in years to come.

Overall, combustion exhaust consists of a complex mixture of particles and gases. Although many studies have addressed links between CGNPs and respiratory or cardiovascular conditions, it is less clear if individuals in at-risk populations (people living in polluted areas) are more likely to suffer neurological damage due to prolonged exposure [20,47,70,71,72,73]. Epidemiological studies around the world have shown correlations between air pollution and brain health. Specifically, brain volume shrinkage and cognitive decline are seen in both children and adults living in polluted regions [20,47,70,71,72,73]. Early Alzheimer’s pathologies have also been observed in healthy subjects living in polluted areas, and air pollution is linked to the risk of PD [29,33]. Even though these epidemiological studies did not specifically consider the CGNP fraction of air pollution, together with our findings, the evidence above clearly suggests that the CGNP fraction of air pollution may be an important contributor to brain health. To our knowledge, we are the first to demonstrate that both AgNPs and CGNPs elicit microglial inflammatory response through an NF-κB mediated pathway. Although we did not screen for pro-inflammatory gene targets regulated by NF-κB, we did demonstrate elevated cytokine levels of TNF-α via ELISA. Given that TNF-α cytokine production is a functional mediator of inflammation, we feel this is a more informative and translatable endpoint.

Interestingly, data suggests that neuromelanin, an endogenous brain compound, has some characteristics that are similar to NPs. Neuromelanin forms protein aggregates which accumulate during the onset of PD, and are linked to neurodegeneration of catecholamine neurons [74]. Neuromelanin can also activate microglia via a NFκB-dependent upregulation of TNF-α and nitric oxide. Furthermore, postmortem brain histology from children living in areas with high air pollution showed brainstem injury associated with elevated accumulation of neuromelanin and α synuclein [75]. A recent study has reported interesting data that demonstrates that N2 murine microglial cells can mount a AgNP-detoxifying mechanism involving the sulfiding of Ag^+^ ions and upregulation of hydrogen sulfide-synthesizing enzymes, potentially representing an endogenous neuroprotective mechanism [76]. Although this study used a different cell model, higher doses of AgNP (50 μg/mL), and a different dosing paradigm, the potential for a protective mechanism is encouraging, highlighting the need for further characterization of the effect of CGNPs on brain health.

Numerous toxicological studies clearly demonstrate that diesel engine emissions has a profound adverse effect on human health [3,13,18,77]. Collectively, data supports that air pollution increases cardiovascular and respiratory disease, and the risk of various cancers. Our data and those of others show that in addition to these known and well-established deleterious effects on health, systemic inflammation, and neuroinflammation caused by air pollution can also result in increased risk for neurodegenerative diseases. Due to the complexity of CGNPs, identifying the toxicity of each individual component still remains a major challenge and highlights the need for future studies. Despite the limitations in our study, our methodology and findings can be adapted to address the growing need for rapid neurotoxic screens of environmental pollutants. The model used here could, in the future, be adapted to assist in the development of high-output genomic screens for microglial and neuronal markers. Given the global impact that air pollution has on public health, research to further elucidate the mechanisms of CGNP-induced neuroinflammation and neurotoxicity through microglial activation is required. Future studies using “omic” approaches, such as next-generation RNA sequencing, proteomics, and metabolomics, are needed to elucidate both the neurological impact and long-term physiological consequences of CGNP exposure, including the risk for the early onset of incurable diseases such as AD and PD [24,33,42,78,79]. Our lab is currently pursuing studies investigating microglial regulation of inflammatory response and rodent models of environmental exposure using real-time diesel exhaust exposure-generated CGNPs to further define targetable neuroinflammatory mechanisms. Our long-term goal is to apply large scale omics technology to compile a comprehensive database of the brain immune response to CGNPs. Once identified, these mechanisms would provide routes for treatment of neurological insults caused by environmental CGNP exposure.
